# Long-Term Stable and Tightly Controlled Expression of Recombinant Proteins in Antibiotics-Free Conditions

**DOI:** 10.1371/journal.pone.0166890

**Published:** 2016-12-01

**Authors:** Soo-Jin Yeom, Yu Jung Kim, Jeongmin Lee, Kil Koang Kwon, Gui Hwan Han, Haseong Kim, Dae-Hee Lee, Hak-Sung Kim, Seung-Goo Lee

**Affiliations:** 1 Synthetic Biology & Bioengineering Research Center, Korea Research Institute of Bioscience and Biotechnology (KRIBB), Daejeon, Republic of Korea; 2 Department of Biological Sciences, Korea Advanced Institute of Science and Technology (KAIST), Daejeon, Korea; 3 Biosystems & Bioengineering, University of Science & Technology (UST), Daejeon, Republic of Korea; Dong-A University, REPUBLIC OF KOREA

## Abstract

Plasmid-based gene expression is a fundamental tool in the field of biotechnology. However, overexpression of genes of interest with multi-copy plasmids often causes detrimental effects on host cells. To overcome this problem, chromosomal integration of target genes has been used for decades; however, insufficient protein expression occurred with this method. In this study, we developed a novel cloning and expression system named the chromosomal vector (ChroV) system, that has features of stable and high expression of target genes on the F′ plasmid in the *Escherichia coli* JM109(DE3) strain. We used an RMT cluster (KCTC 11994BP) containing a silent *cat* gene from a previous study to clone a gene into the F′ plasmid. The ChroV system was applied to clone two model targets, GFPuv and carotenoids gene clusters (4 kb), and successfully used to prove the inducible tightly regulated protein expression in the F′ plasmid. In addition, we verified that the expression of heterologous genes in ChroV system maintained stably in the absence of antibiotics for 1 week, indicating ChroV system is applicable to antibiotics-free production of valuable proteins. This protocol can be widely applied to recombinant protein expression for antibiotics-free, stable, and genome-based expression, providing a new platform for recombinant protein synthesis in *E*. *coli*. Overall, our approach can be widely used for the economical and industrial production of proteins in *E*. *coli*.

## Introduction

Plasmid-based protein production has been an essential tool for several decades in the field of biotechnology because of the ease of construction and ability to transfer the same plasmid into multiple strains [1,2]. In addition, this approach allows for the overexpression of genes of interest, generating large amounts of recombinant proteins [3]. However, an important limitation in the production of recombinant proteins using common plasmids in *Escherichia coli* is the requirement for the selective pressure of an antibiotic to be stably maintained in cells, which is costly for industrial-scale production [4]. The spread of antibiotics and resistance markers may also be unsafe for the environment [4]. Ampicillin is commonly used for plasmid selection and should be avoided in the production of human therapeutic proteins because of the potential for human allergic reactions [5,6].

To overcome these limitations to plasmid-based gene expression, chromosomal integration [7] have been studied because it reduces metabolic burdens and obviates the requirement for antibiotics to ensure plasmid maintenance [8,9]. Tools for the chromosomal integration of DNA have been extensively studied, including CRIM [10], recombineering [11–14], and Tn7-based integration [15,16], and have been successfully implemented in previous studies [7,17]. However, the levels of protein expression produced following chromosomal integration are limited because only a single copy of the gene is present [18]. Furthermore, chromosomal integration method of a target gene may affect essential genes in the cell. Therefore, a new robust cloning system that can be used to express stable target genes at high levels should be developed.

Here, we report a new robust gene expression system named ChroV that enables the direct cloning of target genes into the F′ plasmid in *E*. *coli* JM109(DE3) cells; this method requires no restriction or ligation steps. Based on our previous findings, the ChroV system uses a RMT cloning cassette in a plasmid that contains a silent selection marker activated by the homologous region of target gene PCR products [19]. Here, the *GFPuv* and *crtEBIY* genes were cloned into ChroV-*E*. *coli* JM109(DE3) and their genetic stabilities, expression levels of target proteins without antibiotics, and tight regulation by an inducible promoter were compared to the pRMT system as plasmid versions.

## Materials and Methods

### Materials

*Escherichia coli* JM109(DE3) cells were purchased from Promega (Madison, WI, USA). Arabinose for inducing lambda red recombinase was obtained from Sigma-Aldrich Co. (St. Louis, MO, USA). Chloramphenicol and kanamycin were purchased from Duchefa (Haarlem, The Netherlands). All restriction enzymes and ligases were purchased from New England Biolabs (Ipswich, MA, USA) or Takara (Otsu, Shiga, Japan). The DNA was amplified using Phusion® High-Fidelity DNA Polymerase (New England Biolabs). The Wizard® SV Gel and PCR Clean-Up System from Promega (Madison, WI, USA) were used to purify the PCR products. Oligonucleotides were purchased from Bioneer (Daejeon, Korea).

### Construction of ChroV system

The locus of ChroV was designed to integrate between the *lac*I C-terminus and *lac*A downstream in the *E*. *coli* JM109(DE3) chromosomal DNA by 50 bp homologous regions. The cloning cassette in the pRMT plasmid (KCTC 11994BP, T7 promoter—T7 terminator region) containing the silent cat gene, which encodes a chloramphenicol acetyltransferase missing the start codon, was prepared by PCR (Kan-F: 5′-ggtcgacggatccccggaataatacgactcactataggg-3′, ChroV-R: 5′-tcgctgaacttgtaggcctgataagcgcagcgatcaggcaatttttataatcaaaaaacccctcaagaccc-3′; underline indicates the homologous sequence of *lacA* downstream region in the chromosome). The kanamycin-resistant gene containing a flippase recognition target (FRT) site was prepared by PCR from the plasmid pKD13 (ChroV-F: 5′-cattaatgcagctggcacgacaggtttcccgactggaaagcgggcagtgatgtaggctggagctgcttc-3′, Kan-R: 5′-ccctatagtgagtcgtattaattccggggatccgtcgacc-3′; underline indicates the homologous sequence of *lac*I C-terminus region in the chromosome). The two genes were connected by overlap extension PCR. The *E*. *coli* JM109(DE3) cells harboring the pKD46 plasmid were cultivated at 30°C in LB medium containing 50 mM arabinose to induce the expression of lambda red recombinase and washed 2 times with ice-cold water and suspended in sterile 10% glycerol to prepare electro-competent cells (OD at 600 nm = 100). Electroporation was conducted with 100 ng of the overlap PCR product in 50 μL of competent cells using the Gene Pulser Xcell System (Bio-Rad, Hercules, CA, USA). After electroporation, the cells were reactivated for 1 h at 37°C and the positive clones were selected on LB agar medium containing 10 μg/mL kanamycin. Sequences of the resulting constructs were confirmed by DNA sequencing of the ChroV cassette PCR product. After curing of the pKD46, the kanamycin-resistant gene in the chromosome was deleted using the plasmid pCP20 expressing flippase.

### *In vivo* cloning in ChroV system

To examine the expression level of target protein using the ChroV system, the gene encoding the green fluorescent protein (GFPuv, 0.7 kb) and *crtEBIY* (4 kb, GenBank accession number: GQ149341) were amplified using dual purpose primers for the gene amplification and homologous recombination into ChroV. The forward primer consisted of the homologous region and the 5'-annealing sequence for the *GFPuv* gene (GFPuv-F: 5′-tctagaaataattttgtttaactt taagaaggagatatacatatgagtaaaggagaagaacttttcactgg-3′; underline indicates the homologous sequence of the ChroV in the chromosome), while the reverse primer contained the RBS plus ATG and the 3'-annealing sequence for the target gene (GFPuv-R: 5′-gccattggg atatatcaacggtggtatatccagtgatttttttctc**cat**atgtata***tctcct***tcttatttatttgtagagctcatccatgcc-3′; bold letter and italic bold letter indicate the start codon and RBS respectively, the homologous sequence of the ChroV in the chromosome is underlined). The forward primer for the *crtEBIY* gene consisted of the homologous region and the 5'-annealing sequence (*crtEBIY*-F: 5′-5′- tctagaaataattttgtttaactttaagaaggagatatacatatgaacagtccctctaccactttattgc-3′; underline indicates the homologous sequence of ChroV in the chromosome), while the reverse primers contained the RBS plus ATG and the 3'-annealing sequence for the target gene (*crtEBIY*-R:5′-gccattgggatatatcaacggtggtatatccagtgatttttttctc**cat**atgtata**tctcct**tcttattcacgcccatttgagtgctg-3′; bold letter and italic bold letter indicate the start codon and RBS respectively, the homologous sequence of the ChroV in the chromosome is underlined). One hundred nanograms of the linear DNA was transformed into JM109(DE3)::ChroV cells harboring pKD46 for lambda red recombineering. Positive clones were selected on LB agar medium containing 25 μg/mL of chloramphenicol and validated by colony PCR using a T7 promoter and T7 terminator primers followed by colony fluorescent image analysis (Bio-Rad, Hercules, CA, USA).

### Expression of GFPuv and crtEBIY in ChroV-JM109(DE3)

The JM109(DE3) cells containing ChroV-GFPuv were grown on LB agar plate containing 25 μg/mL of chloramphenicol and 0.5 mM IPTG at 37°C. The colonies images were detected by colony fluorescent image analysis. The colonies were transferred into 50 mL of Luria–Bertani (LB) medium in a 250 mL flask containing 25 μg/mL of chloramphenicol at 37°C, induced with 0.5 mM IPTG at three different optical densities at 600 nm, and cultured 37°C for 12 h. Cultured cells were harvested, washed in PBS (pH 7.4), lysed using cell lytic B buffer (Sigma), and analyzed by SDS-PAGE. The thickness and intensities of GFPuv band were analyzed using an open source image processing program, ImageJ [20]. ChroV-JM109(DE3) cells harboring various gene clusters such as GFPuv (0.7 kb), EGFP fused with a maltose binding protein (2 kb), and carotenoid-biosynthetic pathway gene clusters (4 kb, 6 kb, and 10 kb) as described previously [19], were grown at the same conditions containing 0.5 mM IPTG. These large genes and clusters were selected to facilitate the detection of protein expression based on fluorescence- or color-observation of the resulting colonies. Colonies expressing β-carotene and astaxanthin were yellow and red in color, respectively.

### Monitoring the antibiotics-free expression of GFPuv fluorescence

ChroV-GFPuv (in this study) and pRMT-GFPuv [19] in *E*. *coli* JM109(DE3) cells were cultivated in 50 mL of LB medium in a 250 mL flask without chloramphenicol at 37°C, induced with 0.5 mM IPTG at an OD_600nm_ of 0.5, and grown at 37°C with shaking at 200 rpm for 12 h using multi spin shaker VS-8480MX2-DT (Vision scientific Co.,LTD, Daejeon, Korea). The 5 mL of the grown cell were consecutively transferred to a fresh LB medium (50 mL in a 250 mL flask), once a day for a week to monitor the antibiotics-free protein expression. Cultured cells were harvested, washed in PBS (pH 7.4), and resuspended in cell lytic B buffer from Sigma-Aldrich Co. (St. Louis, MO, USA) for cell lysis. The protein expression was evaluated by SDS-PAGE and GFPuv fluorescence was measured using multilabel plate readers (PerkinElmer, Waltham, MA, USA).

## Results and Discussion

### Construction of ChroV system in *E*. *coli* JM109(DE3)

In previous study, the pRMT system that involves inserting the RBS plus ATG sequence by homologous recombination, resulted in a rapid cloning of PCR-amplified genes in living cells [19]. Although the pRMT system was used successfully for *in vivo* cloning, the expression of target genes still requires the selective antibiotic pressure to maintain the plasmid in cells. To circumvent this issue, we attempted the integration of pRMT into the *E*. *coli* JM109 chromosome at the location of the *bglA* gene, which resulted in a significant decrease of protein expression [19].

**Fig 1:**
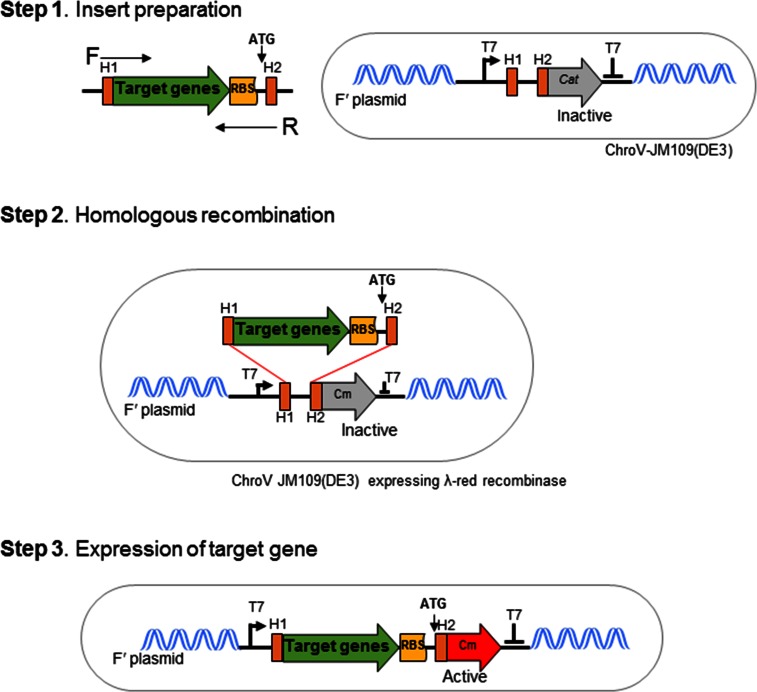
Chromosome vector (ChroV) system in *E*. *coli* JM109(DE3) cells. The scheme of the ChroV-JM109(DE3) system containing the T7 promoter and a silent chloramphenicol resistance gene (*cat*) that can be activated by homologous recombination with target DNA. *GFPuv* gene containing H1 and H2 of an RBS plus ATG sequence was inserted into the Fʹ plasmid in *E*. *coli*, and the *GFPuv* gene was expressed and the chloramphenicol resistance gene was activated.

Thus, in this study we designed the ChroV system as a new method for cloning a gene of interest into the chromosomal DNA. As the specific integrating locus, the *lac* operon in the Fʹ plasmid was selected as a target locus, which has been widely studied and is beneficial for recombinant protein expression [21], because the location can affect the expression level of target genes [22]. The ChroV system constructed by inserting the RMT cassette into the Fʹ plasmid of *E*. *coli* JM109(DE3) (Fig 1). The Fʹ plasmid of *E*. *coli* JM109(DE3) is a circular DNA containing *lac* operon and *proA* and *proB* genes derived from its chromosome [23]. The silent chloramphenicol resistance gene (without ATG) is activated by homologous recombination with the target gene in the Fʹ plasmid (Fig 1). Therefore, the locus of ChroV was designed between the *lacI* C-terminus and *lacA* terminator in the Fʹ plasmid of JM109(DE3)Δ*lac-proAB*. The IPTG induction is not affected as it can be taken up by the lactose permease encoded in *lacY* [24]. Additionally, the TraD36 in the Fʹ plasmid with a mutation eliminating the transfer factor prevents horizontal genetic transfer [25]. Hence, conjugation efficiency of foreign genes into the chromosomal DNA was greatly reduced in our ChroV system. The ChroV-JM109(DE3) clone was confirmed by DNA sequencing and maintained on LB medium containing kanamycin.

### Tightly regulated induction and stable expression of target genes

**Fig 2:**
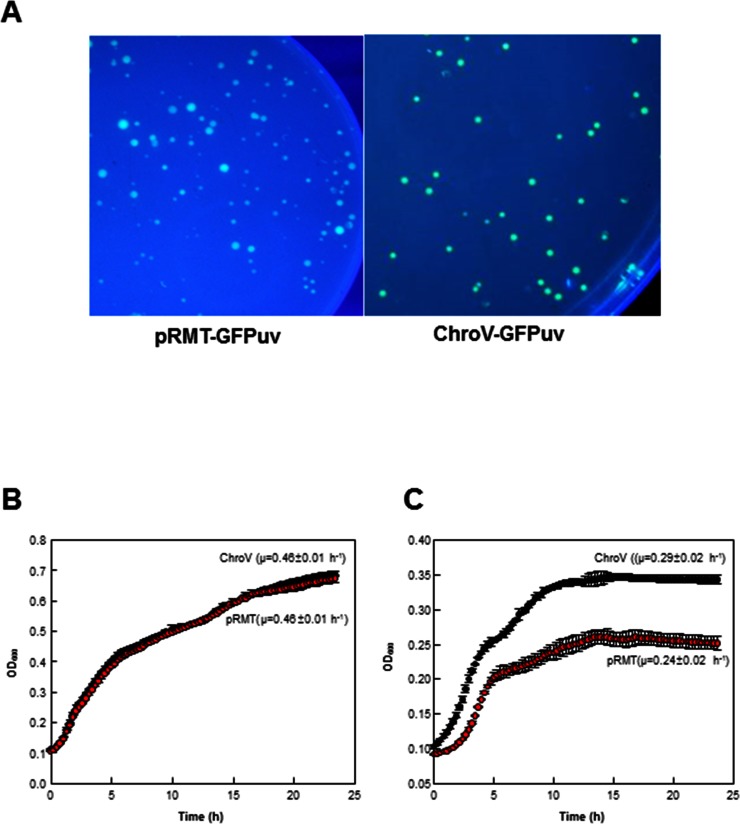
Expression of GFPuv using the ChroV system. A. The *E*. *coli* cells expressing GFPuv in the Fʹ plasmid (ChroV-GFPuv) and GFPuv in the pET plasmid (pRMT-GFPuv) was observed by colony fluorescent image analysis. B. Growth curves of ChroV and pRMT cells in LB media containing 0.5 mM IPTG and 25 μg/mL kanamycin. C. ChroV and pRMT cells were inoculated in M9 media containing 0.5 mM IPTG. The cell growths were monitored in 96 well plates for 24 h at 37°C with shaking at 200 rpm using the Infinite® 200 PRO microplate reader (Tecan Group Ltd, Switzerland) following the manufacturer’s instructions. Error bars indicate a deviation in the triplicate experiments.

**Fig 3:**
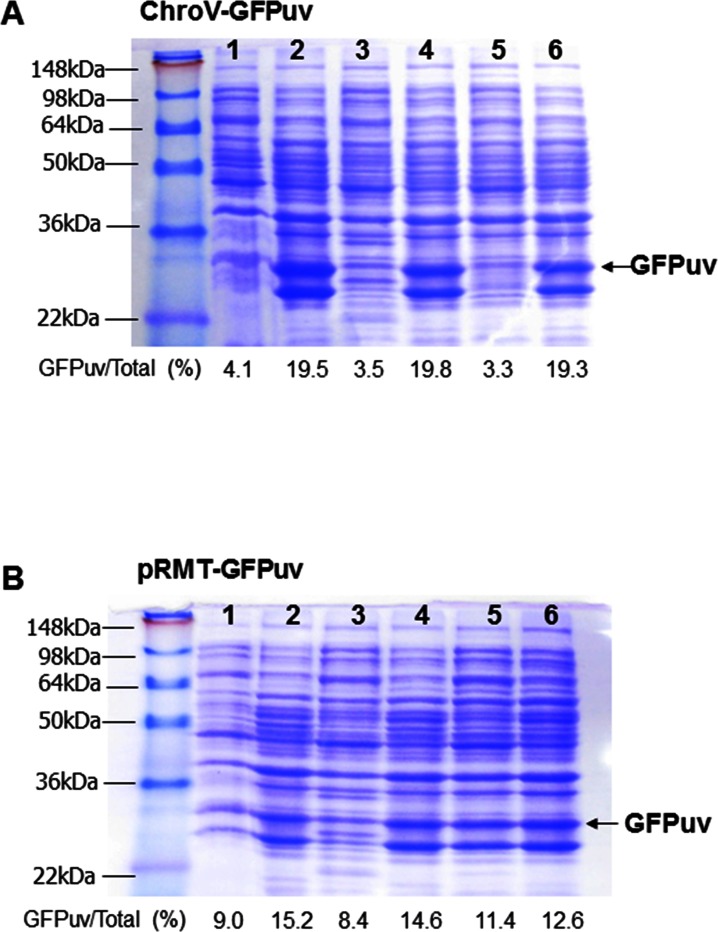
**SDS page analysis of GFPuv expression in ChroV-GFPuv (A) and pRMT-GFPuv (B).** 1: cells of OD_600_ = 0.1; 2: 12 h induction with 0.5 mM IPTG at OD_600_ = 0.1; 3: cells of OD_600_ = 0.5; 4: 12 h induction with 0.5 mM IPTG at OD_600_ = 0.5; 5: cells of OD_600_ = 1; 6: 12 h induction with 0.5 mM IPTG at OD_600_ = 1. The cell lysates were loaded for equal protein concentrations as 5 μg in each lane.

To examine the effectiveness of the ChroV system, a pKD46 variant plasmid containing *GFPuv* gene as the target gene was introduced into the ChroV-JM109(DE3) strain by electroporation. The transformed cells were grown on LB agar medium supplemented with chloramphenicol, indicating the successful activation of the cryptic chloramphenicol resistance gene in the Fʹ plasmid (Fig 2). To check the expression of the GFPuv, we conducted fluorescence image analysis of the GFPuv-ChroV-JM109(DE3) colonies. As a result, colonies expressing the GFPuv in ChroV system exhibited obvious homogeneities of the green fluorescence compared to pRMT-GFPuv cells (Fig 2A). Next, ChroV and pRMT clones were analyzed for the regulation of gene expression by IPTG (Fig 3). The ChroV system was regulated more tightly by an inducible promoter and the expression of GFPuv was not significant in the absence of IPTG (lanes 1, 3, 5 in Fig 3A), whereas pRMT-GFPuv cells expressed a leaky expression at a higher growth level (lane 5 in Fig 3B). Also, the IPTG-induction of the ChroV cells resulted in a high expression of *GFPuv* genes, comparable to the expression level in the pRMT-GFPuv strain despite its low-copy expression (Fig 3), possibly implying the lower metabolic burden in ChroV cells [25]. Indeed, the specific growth rate (μ) of the ChroV-GFPuv cells as 0.29±0.02 h^-1^ was higher than that of pRMT-GFPuv cells as 0.24±0.02 h^-1^ in M9 media (Fig 2B and 2C).

**Fig 4:**
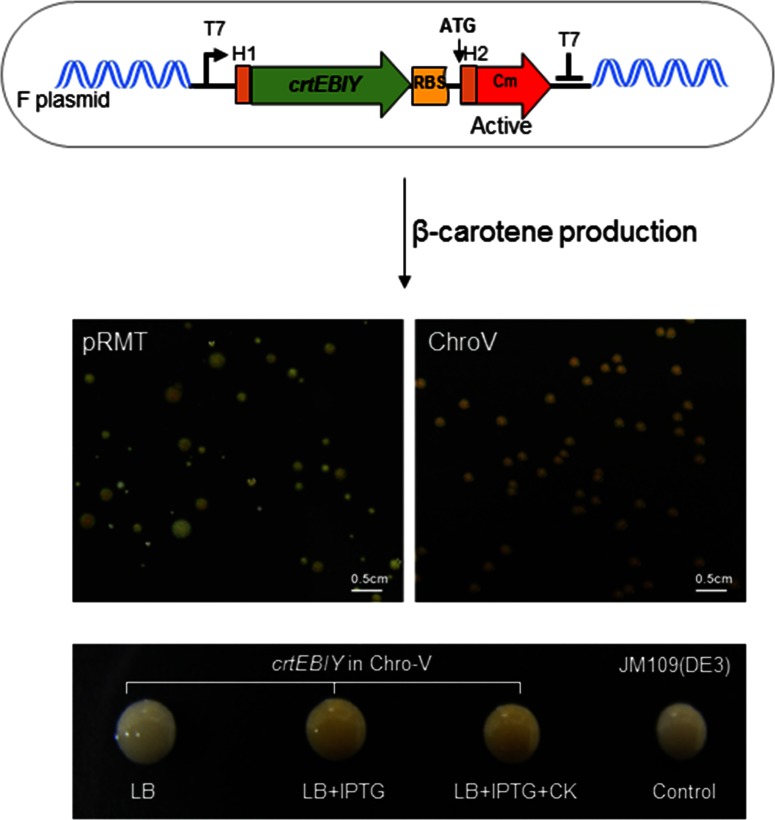
Cloning of the β-carotene synthetic gene cluster in the ChroV system. Shown are *E*. *coli* colonies expressing the β-carotene synthetic genes, which produced yellow-pigmented colonies in the pRMT system [19] and ChroV system (in this study). The ChroV system showed more homogeneous sizes, shapes, and colors for the colonies.

After the validation of ChroV system, we tested the multi-gene system for the cloning of a β-carotene-producing genes, *crtEBIY* (Fig 4). The ChroV system produced more homogeneous colonies compared to those of the pRMT system, which was rather heterogeneous in colony sizes and colors (Fig 4). Moreover, various gene clusters such as GFPuv (0.7 kb), EGFP fused with a maltose binding protein (2 kb), and carotenoid-biosynthetic pathway gene clusters (4 kb, 6 kb, and 10 kb) described in a previous study were recombined into the ChroV system to produce red or yellow color, representing β-carotene or astaxanthin, respectively (S1 Fig). It is particularly notable that the ChroV system produces a clear yellow or red color, indicating that β-carotene or astaxanthin were produced in the absence of antibiotics, showing the antibiotics-free protein expression and fermentation possible.

Chromosomal integration of the gene of interest as powerful alternative may overcome the problems of expression stability in plasmid-based systems and the metabolic burden of plasmid maintenance and replication. Chromosome integration is particularly suitable for metabolic engineering of the host [26]. However, there are several disadvantages such as lower production rates, labor-intensive, time-consuming, and difficulty of determining a suitable position for integration [18,27]. Our ChroV system can be used to overcome the limitations of classical chromosomal integration systems to produce high expression levels of protein, is less time-consuming, and avoids unnecessary insertion into the chromosomal DNA because of the use of the F′ plasmid.

### Stable expression of target gene in antibiotic-free system

**Fig 5:**
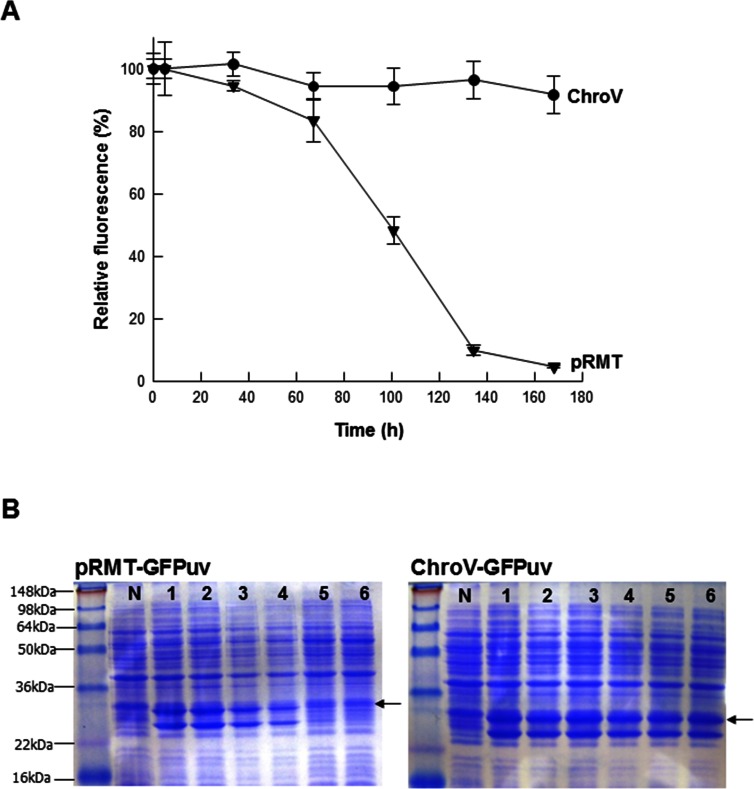
Stable maintenance of GFPuv-expression levels in conditions without antibiotics. A. The expression of GFPuv was analyzed by fluorescent intensity at 510 nm after excitation at 385 nm. B. SDS-PAGE analysis of pRMT-GFPuv and ChroV-GFPuv. N: Negative control. 1: *E*. *coli* culture time for 4 h; 2: culture time for 33 h; 3: culture time for 67 h; 4: culture time for 100 h; 5: culture time for 134 h; 6: culture time for 168 h. GFPuv indicated by arrow.

To prove the stable antibiotics-free expression using the ChroV system, we cultivated two *E*. *coli* strains, ChroV-GFPuv and pRMT-GFPuv, in the absence of antibiotics. 1% of the cells were diluted to fresh media at the specified time-intervals and investigated for the fluorescence expression by IPTG induction (Fig 5A). At seven transfers for six days, the ChroV cells were still observed to express the GFPuv (Fig 5A), while pRMT cells exhibited a gradual decrease in GFPuv expression and eventually no expression. This result was corroborated by SDS-PAGE analysis of pRMT-GFPuv and ChroV-GFPuv (Fig 5B).

It is well-known that the Fʹ plasmid of *E*. *coli* is stable and maintainable in dividing cells [28]. Expression vectors based on the F′ plasmid of *E*. *coli* were constructed and were structurally and segregationally stable for at least 150 generations; they also imposed a low metabolic burden on the cell under conditions of maximum expression of plasmid-encoded β-galactosidase [29]. Moreover, the F factor-based system has been used for the stable inheritance of genetic information, stable expression, and reduced metabolic burden on the host cell [25,30]. To overcome the metabolic burden, antibiotics-free systems based on auxotrophy [31,32] and post-segregational killing systems [33,34] have been evaluated.

Our ChroV system, which takes advantage of the unique features of the Fʹ plasmid, enables homogenous and high expression of target genes without the use of antibiotics. Otherwise, it may be difficult to efficiently produce a protein because some proteins are converted into inclusion bodies or are toxic. In addition, the high expression of the cat gene as a selection marker in the ChroV system may be stressful and detrimental for protein production. Nevertheless, our ChroV system adapted benefits of Fʹ plasmid and RMT system in our laboratory that is possible high genetic stability for protein expression without antibiotic possibly enabling the antibiotics-free production of valuable fermentation products.

## Conclusions

We present a useful method, the ChroV system, for *in vivo* cloning and stable expression as an advancement of our previously described pRMT cloning method [19]. The ChroV system enables a homogenous high expression of target genes. Gene expression levels were comparable to those obtained using plasmid systems. Particularly, the ChroV system showed high genetic stability and inducible, tightly regulated protein expression without antibiotics, and thus it can be used to produce recombinant proteins for long-term culture and antibiotics-free fermentation. Possibly, the ChroV system can also be used in other *E*. *coli* strains containing Fʹ plasmid.

## Supporting Information

S1 Fig Effect of insert size and composition. A. Composition and sizes of various clones in this study. *cat* represents chloramphenicol-resistant gene activated by homologous recombination. B. Images of ChroV-JM109(DE3) colonies expressing various target genes: 0.7, 2kb insert expressed green fluorescence, while 4kb insert produced yellow pigmented colonies. The 6kb and 10kb inserts showed mixed colonies of yellow and red, the specific colors of β-carotenoid and astaxanthin, respectively
(TIF)
