# Supplementary figures and images for: Long-Term Stable and Tightly Controlled Expression of Recombinant Proteins in Antibiotics-Free Conditions
Source: PLoS One. 2016 Dec 1;11(12):e0166890. doi: 10.1371/journal.pone.0166890 (PMC5132264; doi:10.1371/journal.pone.0166890)

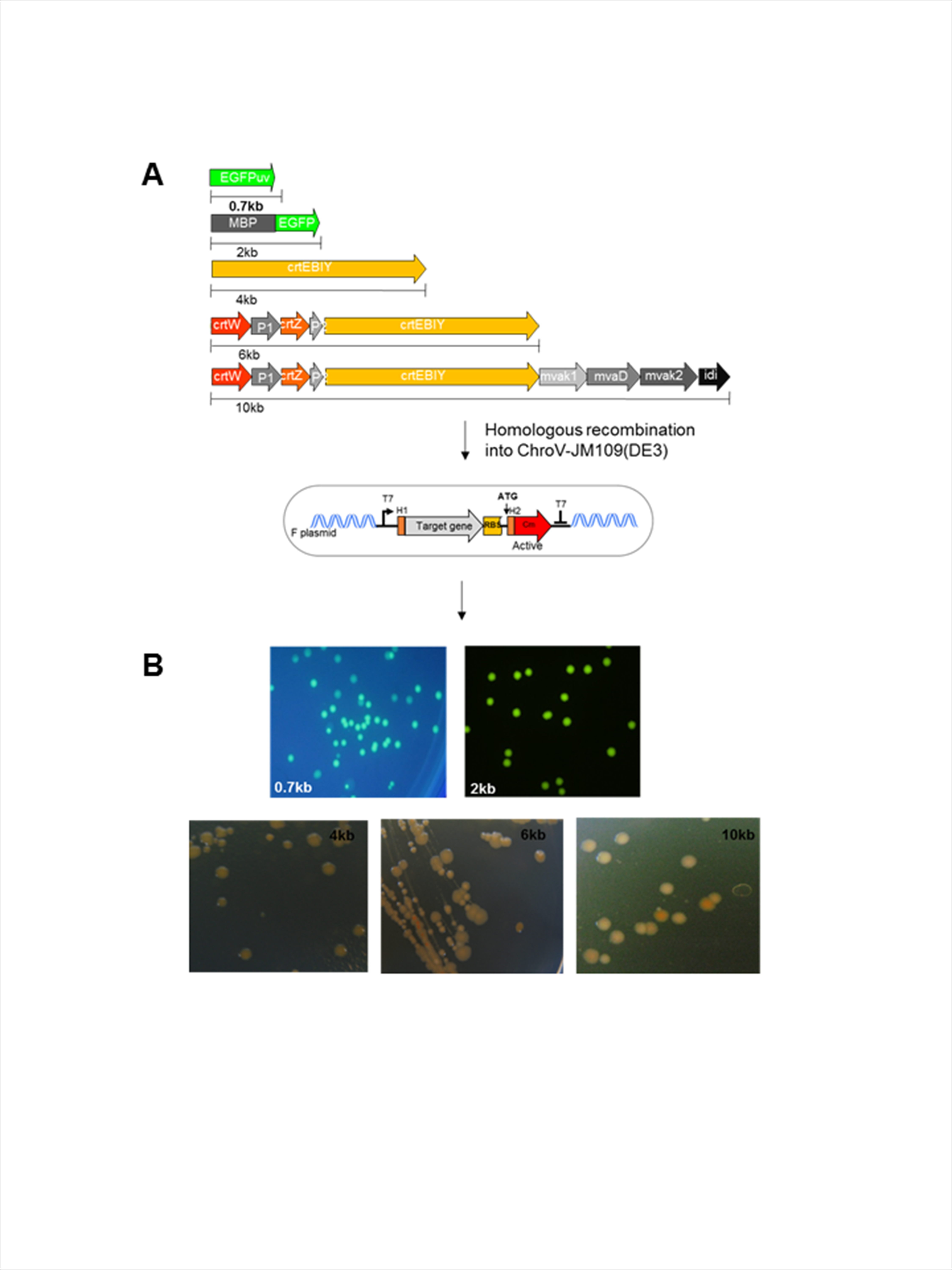

Supplement: S1 Fig — A. Composition and sizes of various clones in this study. cat represents chloramphenicol-resistant gene activated by homologous recombination. B. Images of ChroV-JM109(DE3) colonies expressing various target genes: 0.7, 2kb insert expressed green fluorescence, while 4kb insert produced yellow pigmented colonies. The 6kb and 10kb inserts showed mixed colonies of yellow and red, the specific colors of β-carotenoid and astaxanthin, respectively (TIF) [file pone.0166890.s001.tif]
